# Prey or protection? Access to food alters individual responses to competition in black widow spiders

**DOI:** 10.1093/beheco/araf011

**Published:** 2025-02-06

**Authors:** Tom Ratz, Pierre-Olivier Montiglio

**Affiliations:** Department of Evolutionary Biology and Environmental Studies, University of Zürich, Winterthurerstrasse 190, 8057 Zürich, Switzerland; Département des Sciences Biologiques, Université du Québec à Montréal, CP-8888 Succursale Centre-ville, Montréal, QC, H2X 1Y4 QC, Canada

**Keywords:** among-individual variation, *Latrodectus hesperus*, phenotypic plasticity, resource availability, social interactions, web structure

## Abstract

Animals influence the phenotype and reproductive success of their conspecifics through competitive interactions. Such effects of competition can alter the intensity of selection and ultimately change the rate of evolution. However, the magnitude of the effects of competition, and their evolutionary impact, should vary depending on environmental conditions and individual responses among competitors. We tested whether a key environmental variable, resource availability, affects the response to competition in black widow spiders by manipulating access to prey and the level of competition. We examined if focal spiders modify their web structure and aggressiveness towards prey stimuli when a competitor is present, and whether these responses depend on prior prey access. We also tested if any effects of competition vary with individual differences among competitors. Access to resources changed how individuals respond to competition. Spiders with limited access to prey were less likely to attack prey stimuli in the presence of a conspecific competitor than spiders with greater access to prey, suggesting that limiting resources hinders competitive responses. In contrast, all spiders built better-protected webs in the presence of competitors, regardless of prior access to prey. Crucially, these responses differed among focal spiders and depended on individual competitors. Our findings highlight the importance of environmental conditions and individual differences in mediating the impact of social interactions on phenotypes and eventually on their evolution.

## Teaser

This study examines how resource availability influences individual responses to competitors in black widow spiders. By manipulating access to prey and the presence of competitors, we found that limited resources can reverse how spiders respond to the presence of competitors and reduce individual differences in these responses. These findings emphasize the role of environmental conditions and individual differences in shaping social interactions and their potential evolutionary consequences.

## Introduction

Intraspecific competition plays a key role in shaping the abundance and distribution of species and populations (May 1976; Gurevitch et al. 1992; Bolnick 2001, 2004). However, predicting the effects of intraspecific competition on phenotypic evolution and population dynamics remains challenging because individuals within a population often differ in their responses to changes in the competitive environment (Vellend 2006; Bolnick et al. 2011). For instance, while some great tits increase their aggressiveness as population density increases, others show little or no behavioral change (Araya-Ajoy and Dingemanse 2017; see also Montiglio et al. 2017a for similar findings in water striders). The magnitude of these differences can influence the effects of intraspecific competition on phenotypic evolution, as well as on population and community dynamics (Bolnick et al. 2011; Poisot et al. 2015; Tylianakis and Morris 2017). Therefore, understanding and predicting the ecological and evolutionary effects of competition requires insights into how individuals respond during competitive interactions and how these responses vary with environmental conditions.

Because individuals compete against each other for resources, their responses to competitors often depend on the characteristics of the competitors they encounter, which can shape their behavioral strategies (Moore et al. 1997; Araya-Ajoy et al. 2020). Individual differences in competitive responses should then also depend on standing variation in competitive traits themselves (eg Wilson et al. 2009; Briffa et al. 2015). For example, differences in boldness in male water striders and crickets, which elicits aggressiveness in opponents, explain some variation in aggression during agonistic interactions (Sih et al. 2014; Santostefano et al. 2016). Furthermore, individuals frequently adjust their behavior depending on their competitor’s phenotype. For example, males in frogs, jumping spiders, and paper wasps adjust their aggressiveness levels based on their opponent’s body size, often increasing aggression towards smaller or similarly sized rivals (Wagner 1989; Faber and Baylis 1993; Polak 1994). Thus, the phenotype of competitors, making up the social environment, can influence individual responses to competition. Given that environmental conditions also play a critical role in modulating individual variation in competitive phenotypes and in the responses to competitors (Vellend 2006; Bolnick et al. 2011; Briffa et al. 2015), both the nonsocial *and* social environments might jointly shape the consequences of competition. Interference or exploitative behaviors are costly (Begon and Townsend 2021; Abrams 2022), and variation in resource availability and an individual’s own condition should affect the magnitude of individual differences in competitive traits and in individual responses to competitors. Individuals in poorer conditions may thus lack the energy reserves to sustain their investment in competitive traits. Consequently, the magnitude of individual variation is expected to shift with changes in resource abundance (beyond the effect of resources on competition per se) or any factor altering the acquisition of these resources by individuals (Laskowski et al. 2021; Haave-Audet et al. 2022). However, few studies on competition have investigated individual variation in competitive responses while accounting for condition, physiological state, or the phenotype of competitors.

Here, we address this issue using the western black widow spider *Latrodectus hesperus* as a study system to test whether variation in access to food resources alters the response to competition and the magnitude of individual differences in foraging and defense against competitors. Black widows are ideal to investigate this question given that adult females build a costly web that they adjust in size and structure in response to the availability of prey and past foraging success (Toupin et al. 2022). The web includes structural threads that contribute to protection against predators and intruders, and sticky trap threads that contribute to prey capture (Schraft et al. 2021). Black widows sometimes attempt to take over the web of conspecifics (Blackledge et al. 2003; Krugner et al. 2023) and might adjust the structure of their web and behavior in response to the presence of competitors (Jones et al. 2020). Black widows often build their web near the webs of other conspecifics (Baerg 1923; D’Amour et al. 1936; Trubl et al. 2012) and have occasionally been observed living in groups (Salomon 2007; Bertani et al. 2008; Salomon et al. 2010). Individuals that have more limited access to prey or that are in poorer condition increase the efficiency of their web at catching prey by building a larger number of sticky traps (Blackledge et al. 2005; Zevenbergen et al. 2008; Toupin et al. 2022). In contrast, when access to prey increases, black widows reallocate silk away from sticky traps and invest more in structural threads that provide physical protection against attacks by potential predators and conspecific intruders (Blackledge and Zevenbergen 2007; Jones et al. 2020; Toupin et al. 2022). Black widows also show individual differences in the structure of their web and foraging behavior (DiRienzo and Montiglio 2016a, 2016b; Toupin et al. 2022). There is, however, no information as to whether prey availability and differences among competitors alter how individuals respond to competition and the extent to which individuals differ in the response to competition.

Our aim was to test how variation in prey availability affects: (1) spiders’ adjustments in foraging behavior and web-construction decisions in response to conspecific competitors, (2) the extent of individual differences in foraging and web-construction behavior, and (3) the impact that individual competitors have on focal spiders. To this end, we repeatedly measured aggressive behavior and web construction in female black widow spiders under low or high prey availability, either with or without a conspecific female competitor. If limited access to prey reduces body condition and constrains spiders’ ability for plastic responses–due to the energetic costs of sensory and cognitive adjustments to competition (eg DeWitt et al. 1998; Auld et al. 2010; Snell-Rood 2013)–we expect spiders with lower prey access to invest less in defense against competitors by building less-protected webs. Alternatively, if limited prey access enhances motivation to forage to avoid starvation (Dingemanse and Wolf 2010; Luttbeg and Sih 2010), spiders may respond more aggressively to prey stimuli and build stickier, more effective trap threads under these conditions. Based on prior findings that document individual differences in aggressiveness and web structure (DiRienzo and Montiglio 2016a, 2016b; Toupin et al. 2022), we anticipate that greater prey availability will amplify between-individual differences in these traits via positive feedback mechanisms in resource allocation (Dingemanse and Wolf 2010; Sih et al. 2015). Regardless of prey availability, we expect consistent individual variation in the effects elicited by conspecific competitors, potentially driven by differences in the use of airborne and silk-borne chemical cues that influence conspecific responses (Anava and Lubin 1993; Kasumovic and Andrade 2004). Ultimately, if prey availability increases the magnitude of between-individual differences, it would suggest that traits important for competition are sensitive to variation in the biotic environment. Conversely, the absence of such effects would indicate that these traits are robust to environmental variation. By analyzing competitive phenotypes at both the between- and within-individual levels, we aim to elucidate how the social and nonsocial environments jointly shape the outcomes of intraspecific competition.

## Materials and methods

### Origin and maintenance of the spiders

We used female black widow spiders collected as adults in July 2021 in Davis, California, United States. We used females because males invest less in web construction and abandon it to seek reproductive partners upon maturity (Sergi et al. 2020). We transferred spiders to the University of Quebec at Montreal, Quebec, Canada, and kept them for 2 mo under standardized laboratory conditions (23 °C, 25% humidity, and a 12-h photoperiod) in individual transparent plastic containers (946 mL). We fed spiders a single live house cricket (*Acheta domesticus*) every 2 wk before the start of the experimental food treatments. Two months prior to the experiment, we initiated the food treatments. We assigned 18 females to a low food treatment, providing one cricket every 4 wk, and 18 females to a high food treatment, providing two crickets every 4 wk. These feeding frequencies reflect the range of prey availability typically encountered by black widows in the wild (Johnson et al. 2012), while minimizing the effects on survival, which decreases when individuals receive fewer than one prey item per month (Forster and Kavale 1989).

### Experimental design

To investigate whether access to food influences how female black widows respond to the presence of a conspecific female, we manipulated feeding frequency and allowed experimental females to build three consecutive webs either in the absence or presence of a stimulus female. This number reduces the cost of web building on individuals (Blackledge and Zevenbergen 2007; Zevenbergen et al. 2008), while allowing to assess individual variation in web building (eg DiRienzo and Montiglio 2016a, 2016b). Focal spiders that built their first web in the presence of a stimulus spider built a second web in the absence of a stimulus spiders, and vice versa. To prevent confounding effects of the order of past treatments during previous round of web construction (ie stimulus spider present–absent or absent–present), we haphazardly allocated stimulus spiders among focal spiders for the third round of web construction. Hence, half of the focal spiders were assessed twice in presence of a stimulus spider and once alone, while the other half was assessed once in presence of a stimulus spider and twice alone.

### Web assays

To assess web structure, we quantified the proportion of structural threads relative to sticky trap threads produced at the end of a web construction assay. Before each assay, we weighed focal spiders and moved them in an individual standardized cardboard frame (31 × 17 × 24 cm) placed in a 66 × 41 × 34 cm plastic container sealed with a lid, following established protocols (eg DiRienzo and Montiglio 2016a, 2016b). These frames included on one of the top edges a shelter made from a half-cut section of an egg carton, which all spiders used as their hide. We randomly allocated half of the focal spiders a stimulus adult female spider from the same population, kept on an ad libitum food regime (ie one cricket every 2 wk). We weighed the stimulus spiders and moved them into an individual frame, next to the frame of the focal spider within the same plastic container. We confined the cardboard frame containing the stimulus spider in a mesh cage to prevent any direct interactions between the focal and stimulus spiders. Black widows detect the presence of a conspecific in the close vicinity using airborne chemical cues that are transmitted in the air (Anava and Lubin 1993; Kasumovic and Andrade 2004) and increase their investment in silk production when building a web within a short distance from that of a conspecific (<1 m; Salomon 2007). For the first round of web construction, 9 spiders from the low-food access treatment were alone, 9 in presence of a stimulus spider, 9 high-food access spiders were in presence of a stimulus spider, and 9 were alone. We kept all spiders in the same temperature, humidity, and light conditions described above.

Focal and stimulus spiders built their web for 7 d, which is enough time to build a full web (eg Blackledge and Zevenbergen 2007; Toupin et al. 2022). At the end of the 7-d period, we removed all spiders from their web construction frame, weighed them to record body mass after web construction and put them back in their original individual containers for 3 wk to recover before the next round of web construction on their original food treatment (ie low- or high-food access). We repeated this procedure for a third round of web construction. To test whether individual differences in stimulus spiders influenced the response of focal spiders, we used the same 18 individuals as stimulus spiders for the three rounds of web construction. Thus, each stimulus spider built webs in presence of three different focal spiders.

At the end of each round of web construction, we counted the number of sticky trap threads (threads with glue droplets connecting the web sheet to the bottom of the frame) and structural threads (any other threads connecting the web sheet to the bottom of the frame) spun by both focal and stimulus spiders. We used the proportion of structural threads over sticky trap threads as a measure of web structure indicating relative investment in protection versus prey capture (Blackledge and Zevenbergen 2007; Zevenbergen et al. 2008; DiRienzo and Montiglio 2016a, b). We also collected each web to measure the wet mass of silk to the nearest microgram. All web-related measurements (the number of threads and silk mass) were recorded blindly with respect to the treatment group.

### Aggression tests

We assessed the level of aggressiveness of focal spiders on days 5 and 6 after spiders had started building their second and third webs. We used a standardized vibrating stimulus that mimics the vibrations produced by a prey trapped in the web (eg DiRienzo et al. 2013; DiRienzo and Montiglio 2016b). We used a 5-cm-long plastic cable tie attached to a vibrating system (Cnhidee vibrator), which allowed us to apply vibratory cues (approximately 1-s-long pulses at 100 cycles/s separated by 0.5-s-long periods with no pulses; Vibert et al. 2014; DiRienzo and Montiglio 2016a; DiRienzo et al. 2019) on a single silk thread at given locations of the web. Pilot tests confirmed that spiders can detect and respond to this stimulus by rapidly running towards the source of the vibrations and attempted to cover it with silk as they would with insect prey. The vibratory stimulus was applied for 10 s at three different locations: near the shelter (within 2 cm), at the farthest point from the shelter, and approximately midway between these two points. Applying the vibratory stimulus in multiple locations minimizes bias due to the specific application point and reduces the risk of the spider failing to detect the vibrations, such as when the stimulus is applied to an unconnected thread. We waited 10 s between trials if the spider did not respond, or 10 s after the spider retreated back to the shelter if it responded (DiRienzo and Montiglio 2016a, b; DiRienzo et al. 2019). To ensure that the order in which the stimuli were applied (near, far, between) did not influence the behavior of the spider, we ran the three trials for each spider in a random order. Series of three trials were run twice per day (once in the morning and once in the afternoon) on two consecutive days (ie day 5 and day 6) during two web construction assays. We therefore ran 8 series of three trials on each spider (n = 24 trials per individual spiders).

We recorded the level of aggressiveness towards the vibratory stimuli using an ordinal score ranging from 0 to 4. We gave a score of 0 when the spider did not leave the shelter at the end of the 10-s trial, 1 when the spider left the shelter but traveled less than half the distance between the shelter and the stimulus, 2 when the spider traveled approximately half the distance, 3 when the spider traveled more than half the distance, and 4 when the spider reached the stimulus.

### Statistical analysis

We conducted all statistical analyses using R version 3.6.0 (R Development Core Team 2019) loaded with the packages *car* (Fox and Weisberg 2019), *MASS* (Venables and Ripley 2002), *DHARMa* (Hartig 2017), *ggplot2* (Wickham 2016), *lme4* (Bates et al. 2015), *glmmTMB* (Brooks et al. 2017), and *rptR* (Stoffel et al. 2017). We verified the assumptions of normality of residuals and heteroscedasticity using diagnostic plots. For models fitted with Poisson or binomial error distributions, we assessed overdispersion and accounted for it when necessary.

We assessed how the food treatment and the presence of a stimulus spider affected body mass and the investment in web construction. We analyzed data on body mass before web construction using linear mixed models with the food treatment (low- or high-prey access) as sole fixed effect and the identity of the focal spiders as random effects. We used a linear mixed model to analyze data on the change in relative body mass over web construction (hereafter weight loss) and web mass. Weight loss was first square-root and cosine transformed to approach Normal distribution and shifted to strictly positive values by adding the maximum value +1 to all data values. We included as fixed effects the food treatment, the presence of a stimulus spider (present or absent) and the interaction between the two, and as random effects the identity of focal spiders. Repeated measures correlations (rmcorr) revealed a positive link between body mass and investment in silk production (Fig. S1), reflecting that larger spiders produced more silk (DiRienzo and Montiglio 2016a, 2016b). We thus included body mass before web construction, the interaction between body mass and the food treatment, and the interaction between body mass and the presence of a stimulus spider as additional fixed effects in the model analyzing web mass. We analyzed the total number of threads produced using a generalized linear mixed model assuming a Poisson error structure and including as fixed effects the food treatment, stimulus spider presence, the interaction between the two, and focal ID as a random effect. We also included an observation-level random effect (OLRE) to control for overdispersion (Harrison 2015).

Next, we assessed how the food treatment and the presence of a stimulus spider affected the responses to competition. To ease the interpretation of the analysis output, we modeled aggressiveness scores (ranging from 0 to 4) as a binomial-distributed constrained count using a binomial generalized linear mixed model, and included as fixed effects the location of the vibratory cues (near, far, between), the food treatment, the stimulus spider presence, and the interaction between the food treatment and stimulus spider presence. We included the trial number as an additional fixed effect to account for potential habituation or sensitization across trials. To handle pseudoreplication and overdispersion, we included focal ID, trial series, and observation level as random effects.

We analyzed the proportion of threads that were structural threads using a binomial generalized linear mixed model including food treatment, stimulus spider presence, and the interaction between the two as fixed effects, and focal ID as a random effect. We then assessed the effects of the food treatment on individual differences in foraging and competitive traits. To this end, we ran separate models for spiders in the high-food and low-food treatments and estimated adjusted repeatability (the proportion of among-focal variance relative to total variance) on the original scale for aggressiveness towards prey stimuli and web structure. To assess whether repeatability was significantly greater than zero, we used p-values from likelihood ratio tests (LRT) performed by the *rpt* function, which compare fully parametrized models including the individual random effect to models excluding it, using one degree of freedom (Self and Liang 1987; Pinheiro and Bates 2000). We also compared variances explained by focal ID between the two sets of models using unstandardized variance estimates and 83% confidence intervals around these estimates (Royauté and Dochtermann 2021). Note that these models were similar to the ones described above, except that food treatment was not included as fixed effect.

Finally, we assessed the proportion of individual variance in focal aggressiveness and focal web structure due to differences among individual stimulus spiders. We calculated repeatability estimates on the original scale using models with the same structure as those analyzing aggressiveness and the proportion of structural threads. These models included the identity of the stimulus spider as an additional random effect and excluding data from webs built without a stimulus spider. As previously described, we assessed the significance of repeatability estimates using likelihood ratio tests (LRT) that compared models with and without the individual random effect. We then assessed the effect of the structure of the web of stimulus spiders (ie the proportion of structural threads versus sticky trap threads) on the web structure of focal spiders by comparing the proportion of variance explained by the stimulus spider ID random term in the model analyzing focal web structure relative to that of a model including the web structure of the stimulus spider as an additional fixed effect.

## Results

### Effects of food treatment on body mass and investment in web construction

Spiders with low access to food were on average 20% lighter at the onset of the experiment than spiders that had high access to food (mean body mass: low-access = 0.253 g; high-access = 0.317 g; χ^2^ = 7.39, df = 1, p = 0.006), confirming that our food treatment had the intended effect of reducing the mass of low-food spiders. All spiders lost weight during web construction, with spiders from the high-food access treatment losing relatively more weight than spiders from the low-food treatment (mean weight loss: low-access = −8.88%; high-access = −13.7%; χ^2^ = 4.31, df = 1, p = 0.037). Weight loss during web construction was unaffected by the presence of a stimulus spider (χ^2^ = 0.156, df = 1, p = 0.692) or the interaction between the food treatment and the presence of a stimulus spider (χ^2^ = 0.299, df = 1, p = 0.584).

**Fig. 1.**
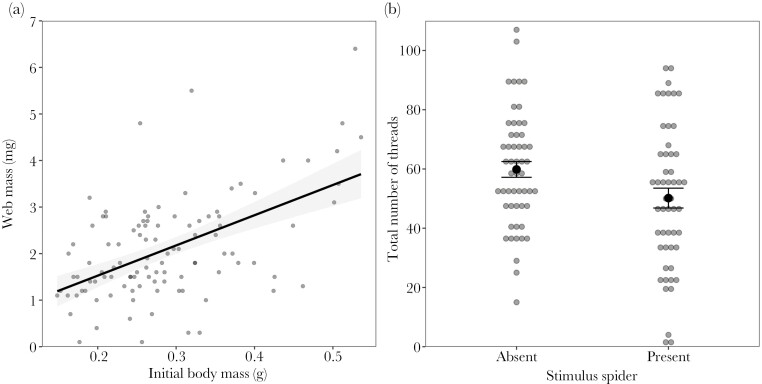
Effects of (a) initial body mass on web mass and (b) the presence of a stimulus spider on the total number of threads in webs built by focal spiders. The solid line represents a linear regression, black dots with error bars indicate means ± standard errors, and gray circles represent raw data. Data were collected from 36 spiders over three rounds of web construction (except for two spiders that built one and two webs, respectively).

Web mass did not vary with the presence of a stimulus spider (χ^2^ = 2.59, df = 1, p = 0.107), nor with food treatment (χ^2^ = 0.037, df = 1, p = 0.845), or their 2-way interaction (χ^2^ = 0.627, df = 1, p = 0.428). Spiders that were initially heavier produced webs with more silk (Table S1). Web mass increased with body mass and this effect was amplified in the presence of a stimulus spider (2-way interaction: χ^2^ = 4.51, df = 1, p = 0.033; Fig. 1a), but unaffected by the food treatment (χ^2^ = 0.015, df = 1, p = 0.901).

Focal spiders in the presence of a stimulus spider overall produced fewer threads (χ^2^ = 7.11, df = 1, p = 0.007; Fig. 1b; Table S1). The total number of threads did not differ between the food treatments (χ^2^ = 0.390, df = 1, p = 0.532), nor did it vary with initial body mass (χ^2^ = 0.421, df = 1, p = 0.516), and there was no significant effect of the interaction between the food treatment and the presence of a stimulus spider (χ^2^ = 1.07, df = 1, p = 0.300).

### Effects of the food treatment on the responses to competition

**Fig. 2.**
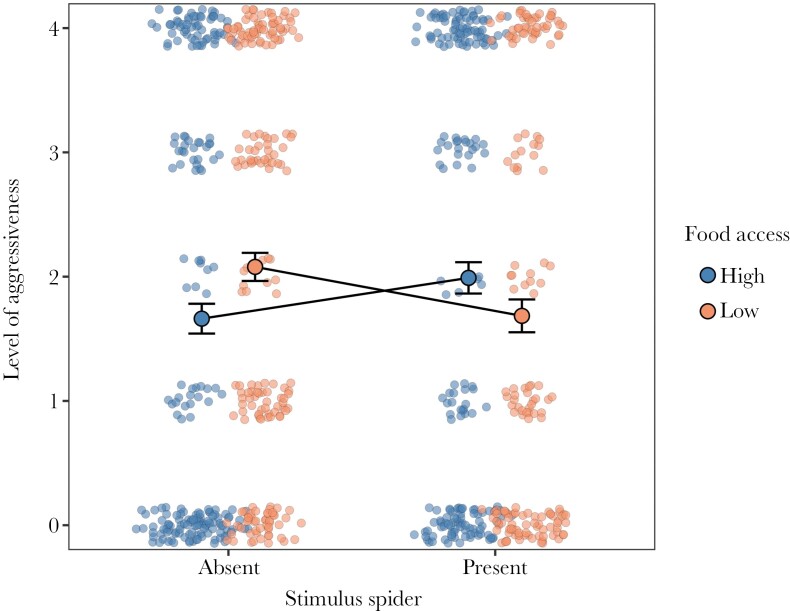
Effects of food access (high or low) and the presence of a stimulus spider on the aggressiveness of focal spiders. Aggressiveness was measured as the rapidity to attack a standardized prey stimulus during the 10-s trial period (see main text for full details on how aggressiveness was scored). Dots with error bars represent means ± standard errors. Circles in dull color represent raw data points. A jitter function was added to separate overlapping values. Data were obtained from 792 observations from 35 spiders each tested on two different webs (except for three spiders tested on a single web).

We found a significant effect of the interaction between the food treatment and the presence of a stimulus spider (Table S2), indicating that focal spiders from the low-food treatment reduced their aggressiveness towards a prey stimulus when they were in presence of a stimulus spider, whereas focal spiders from the high-food treatment increased their aggressiveness in the presence of a stimulus spider (Fig. 2). The location of the vibratory cues had a significant effect (χ^2^ = 12.6, df = 2, p = 0.001), reflecting that spiders were more responsive to near and intermediate stimuli relative to far stimuli (Table S2). The trial number also had a negative effect on aggressiveness (χ^2^ = 21.8, df = 1, p < 0.0001, Table S2), indicating that spiders became less responsive to prey stimuli over time and suggesting habituation.

**Fig. 3.**
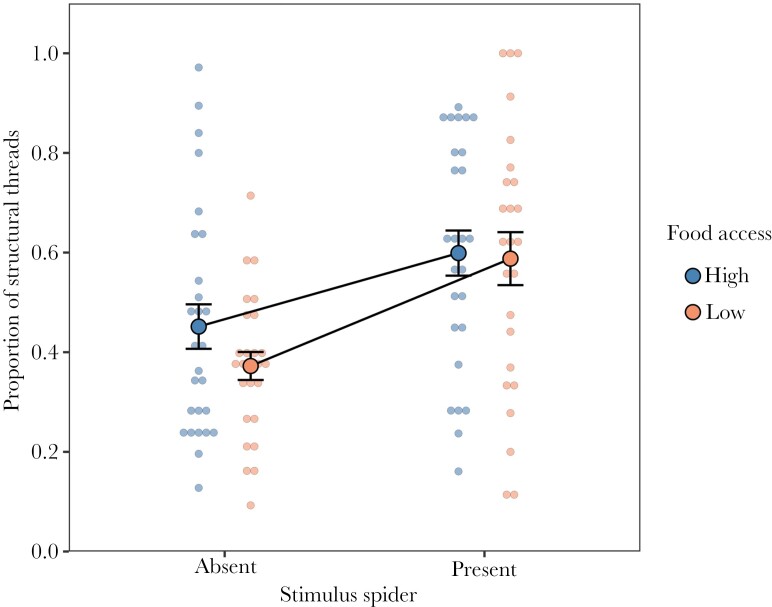
Effects of food access (high or low) and the presence of a stimulus spider on the proportion of structural threads relative to sticky trap threads. Dots with error bars represent means ± standard errors. Circles in dull color represent raw data points.

There was no effect of the food treatment (χ^2^ = 1.18, df = 1, p = 0.276) but a significant effect of the presence of a stimulus spider (χ^2^ = 17.8, df = 1, p < 0.0001) on the proportion of threads that were structural. This effect reflects that spiders built relatively more structural threads in the presence of a stimulus spider (stimulus spider present−absent: estimate = 0.640, SE = 0.086, z = 7.43, p < 0.0001; Fig. 3). Food treatment and the presence of a stimulus spider did not interact to affect the proportion of structural threads (χ^2^ = 0.275, df = 1, p = 0.600).

### Effects of the food treatment on individual differences in foraging behavior and web construction

We then tested whether prior access to prey affected individual differences in how focal spiders respond to the presence of a stimulus spider. We found that differences among individual focal spiders in aggressiveness were repeatable and accounted for 25.4% of the total variance for spiders on the high-food treatment and 33.6% for spiders on the low-food treatment, while there was no difference in repeatability between the two food treatments (high-food treatment: repeatability = 0.254, 95% CI = 0.112 to 1.08, P < 0.0001; low-food treatment: repeatability = 0.336, 95% CI = 0.089 to 0.729, P < 0.0001). However, the variance in aggressiveness due to the focal spider identity was greater for the high-food treatment relative to the low-food one (high-food treatment: variance = 9.60, 83% CI = 5.57 to 16.54; low-food treatment: variance = 2.61, 83% CI = 1.40 to 4.85). We found that differences among individual focal spiders in the proportion of structural threads were repeatable and accounted for 6.4% of total variance for spiders on the high-food treatment (repeatability = 0.064, 95% CI = 0.000 to 0.141, P = 0.027), while we found no evidence that these differences were repeatable for spiders on the low-food treatment (repeatability = 0.035, 95% CI = 0.000 to 0.088, P = 0.085). Variance in the proportion of structural threads due to the focal spider identity did not differ between the food treatments (high-food treatment: variance = 0.344, 83% CI = 0.087 to 0.773; low-food treatment: variance = 0.176, 83% CI = 0.000 to 0.460).

### Effects of the phenotype of stimulus spiders on the responses to competition

Our final analyses aimed at testing if differences among individual stimulus spiders affected the level of aggressiveness in focal spiders and how focal spiders adjusted the structure of their web to competition. We found that differences among stimulus spiders explained 7.04% of the variation in focal aggressiveness (repeatability = 0.070, 95% CI = 0.000 to 0.529, P = 0.020), and 10.7% of the variance in the proportion of structural threads of focal spiders (repeatability = 0.107, 95% CI = 0.009 to 0.217, P = 0.006). Further, we found that the proportion of structural threads built by stimulus spiders had a significant effect on the proportion of structural threads built by focal spiders (χ^2^ = 4.46, df = 1, p = 0.034). A comparison between models with and without the inclusion of the web structure of stimulus spiders showed that incorporating this variable significantly improved model fit (χ² = 4.06, df = 1, P = 0.043). Differences among stimulus spiders in the proportion of structural threads explained 27.6% of the variance in the web structure of focal spiders. These results show that focal spiders adjusted their web structure to differences among stimulus spiders, including differences in the web structure of stimulus spiders.

## Discussion

We found that black widow spiders adjust their investment in the web and its structure in response to competition. The presence of a competitor also affects foraging behavior, with this effect reversing depending on food abundance: higher access to prey was associated with an increased response to prey stimuli in the presence of a competitor and overall greater individual differences in this response. Stimulus spiders vary consistently in the response that they elicit in focal spiders, partly as a function of the structure of their web. Our findings indicate that variation in environmental conditions–food abundance and phenotypic differences among interacting conspecifics–affects individual responses to intraspecific competition. Below, we discuss the broader implications of these findings to our understanding of the effects that competition, and more generally social interactions, can have on phenotypes and their evolution.

### Responses to intraspecific competition

Our finding that access to food influences behavioral responses to competition in terms of aggressiveness, but not web architecture, suggests that individuals with restricted food access or in worse condition may have limited ability to increase aggressiveness. This is perhaps not surprising given that responding to competition is costly (eg Relyea and Auld 2005; Nandy et al. 2016), and thus environmental conditions that influence resource acquisition might often affect the ability of organisms to respond to competitors. For example, in the Argentine ants (*Linepithema humile*), colonies that have more limited access to key nutrients and food resources show reduced foraging activity and aggression against conspecific competitors (Grover et al. 2007). Our study provides further evidence suggesting that environmental conditions, by affecting individual state, are key determinants of the effects of competition on the phenotype and fitness of organisms (Bolnick et al. 2003, 2011; Berg and Ellers 2010).

Environmental conditions could also change the way individuals use shared resources (eg Formanowicz 1982; Inoue and Matsura 1983; Sirot 2000; Turner 2004). For example, increasing the availability of nutrients reduces the negative effects of intraspecific competition on growth rate in the freshwater snail *Helisoma trivolvis* (Turner 2004). This is, however, unlikely to account for our findings given that spiders in our study did not compete for the same prey. Instead, our findings are consistent with the idea that increasing foraging effort, in this case by increasing prey attack rate, can be costly. Spiders on the low food treatment were on average 20% lighter than well-fed spiders, and reduced body condition might limit the ability to cope with the costs associated with mounting a behavioral response (Snell-Rood 2013). If such a reduced response negatively impacts prey capture, we should further expect spiders with limited access to prey to incur greater detrimental effects of competition compared with spiders that had access to more prey. In accordance with this idea, there is a rich literature on condition-dependent foraging (Moran et al. 2021), which also suggests that predators often base their decision to attack a prey depending on their own state (eg Eltz 1997; David et al. 2012; Lyon et al. 2018). For example, ant-lion larvae that suffer limited access to prey build smaller, less costly pits to trap their prey compared with larvae that have had access to more prey (Eltz 1997). Thus, access to food might alter an individual’s condition, ultimately affecting the decision to forage or compete with conspecifics (Rands et al. 2004; Lihoreau et al. 2015). Interestingly, our results revealed that the abundance of prey and the presence of competitors interacted to shape a spider’s decision to attack prey but did not affect web structure. This further suggests that decisions to forage and engage in competition are sometimes interconnected and at other times independent, depending on the type of trait under scrutiny.

Given that conspecific intruders can take over a spider’s web, and assuming that structural threads protect against such take overs, the greater number of structural threads spun by spiders in the presence of a competitor suggests that black widows increased protection against takeovers when in the presence of a conspecific, irrespective of prior access to resources. This result is in line with the findings of previous studies in other taxa showing that spiders adjust the architecture of their web to variation in the intensity of competition with conspecifics and other species (eg Gillepsie 1987; Kennedy et al. 2019). We suggest that spider webs, which are at the interface between the spider and its environment, provide a particularly useful system to study the interplay between environmental variation and intraspecific competition.

### Individual differences in the responses to intraspecific competition

As predicted, differences in aggressiveness among individuals were stronger for spiders on the high-food treatment relative to spiders on the low-food treatment. This difference was detectable despite our modest sample size (36 individuals), denoting a relatively large effect size (Martin et al. 2011; van de Pol 2012). This pattern is consistent with previous research reporting greater among-individual variance in behavior under high food availability or high-quality diets in this and several other systems (DiRienzo and Montiglio 2016a; Han and Dingemanse 2017; Royauté and Dochtermann 2017). Overall, our findings confirm that variation in environmental conditions that affect individual condition, such as food availability, can contribute to variation in traits important for competition. A positive effect of resource availability or quality on between-individual differences is generally expected when individuals vary in their resource acquisition and/or allocation, and when such initial differences are exacerbated through a positive feedback in environments with more abundant resources (Dingemanse and Wolf 2010; Sih et al. 2015). This state-behavior feedback has been predicted by conceptual and theoretical studies (Biro and Stamps 2008; Mathot and Dall 2013; Sih et al. 2015), but it remains rarely tested empirically. Our study constitutes one such test that further suggests that resource abundance can drive a state-behavior feedback. Given the importance of individual differences in competition (Bolnick 2003; Bolnick et al. 2011), we encourage future work to analyze how variation in resource availability might also impact competitive interactions indirectly by altering the extent to which competitors differ from one another.

### Individual differences in the effects of competitors

Differences among individual stimulus spiders also contributed to variation in the web structure of focal spiders: over one-quarter of this variation was caused by differences in the structure of the stimulus spider’s web. Consistent behavioral differences among individuals might explain why they vary in the response that they elicit (Dingemanse and Wolf 2013; Jeanson and Weidenmüller 2014; Stamps 2016). Differences in how individuals impact social partners is an important source of phenotypic variation within a population (Araya-Ajoy et al. 2020; de Groot et al. 2023) that can shape the structure of social groups (Schürch et al. 2010; Aplin et al. 2013; Jolles et al. 2017). For instance, individual differences in social impact can lead to the emergence of so-called “keystone individuals” that have disproportionately large effects on their social partners (Cote et al. 2010; Chang and Sih 2013; Modlmeier et al. 2014). This is the case, for example, in the stream water strider *Aquarius remigis* where larger and more active and aggressive individuals have disproportionate impact on the phenotype of competitors and sexual partners by greatly limiting the mating activity of other members of their group (Montiglio et al. 2017b; Perez et al. 2019). Our results reveal that social partners can impact the phenotype of a focal individual in different ways, even in the absence of physical contact. Future work could explore whether such impact arising from indirect, distant interactions can lead to the emergence of keystone individuals in social networks.

## Conclusions

By revealing that variation in both food abundance and in the phenotype of competitors influences individual responses to competition, our study establishes that the biotic and social environments can shape the expression and variation of traits important in competitive interactions, such as foraging and defense against competitors. The social environment is an important source of phenotypic variation within a population (eg Webber and Vander 2018; Levengood et al. 2022). We suggest that social interactions among phenotypically different conspecifics can be a mechanism maintaining phenotypic diversity within a population. In general, the local environment and the magnitude of among-individual differences might often be important determinants of the broader consequences of competitive interactions on populations and communities.

## Supplementary Material

araf011_suppl_Supplementary_Figure_S1_Tables_S1-S2

## Data Availability

Analyses reported in this article can be reproduced using the R code and data provided by Ratz and Montiglio (2025).
