# Supplementary material for: Prey or protection? Access to food alters individual responses to competition in black widow spiders
Source: Behav Ecol. 2025 Feb 6;36(2):araf011. doi: 10.1093/beheco/araf011 (PMC11851065; doi:10.1093/beheco/araf011)
Supplement: araf011_suppl_Supplementary_Figure_S1_Tables_S1-S2 [file araf011_suppl_supplementary_figure_s1_tables_s1-s2.docx]

**Supplementary information**

We calculated repeated measures correlations (rmcorr) between pairs of traits, which accounts for non-independence among observations (Bakdash and Marusich 2017). Not surprisingly, we found that the total number of threads was positively correlated with both the number of structural threads and the number of sticky trap threads (Figure S1). There was also a large positive correlation between body mass and web mass, indicating that heavier spiders produced more silk during web construction compared with lighter spiders. There were also negative correlations between body mass and the number of sticky trap threads, and between body mass and relative weight loss during web construction, indicating that heavier spiders put less emphasis on prey-capture and lost relatively more weight compared with lighter spiders. Additionally, there was a large negative correlation between weight loss and web mass, indicating that spiders that produced heavier webs lost more weight.

***
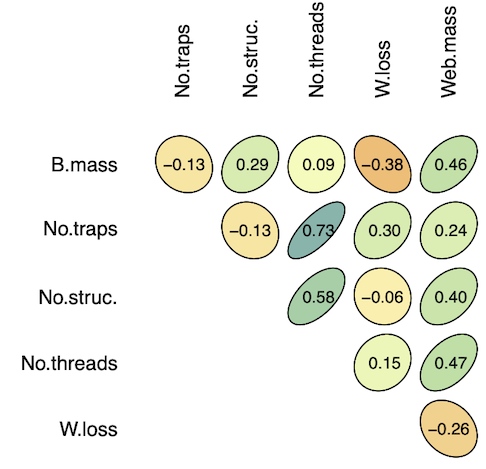
***

Figure S1. Matrix of repeated measures correlations among morphological traits and web traits. Initial body mass (B.mass), number of sticky trap threads (No.traps), number of structural threads (No.struc.), total number of silk threads (No.threads), weight loss during web construction (W.loss), and total web mass (Web.mass). Data were obtained from 36 spiders. Each spider was measured over the course of 3 rounds of web construction, except for two spiders that died before the 3rd round and for which only data from the 1st and 2nd rounds were collected. The figure was made using the ggpairs function of the R package *GGally* (Schloerke et al. 2018)

**Table S1:** Determinants of web mass and the total number of threads in the web (N = 108 webs from 36 focal spiders).

|  | Web mass | | | |  | Total number of threads | | | |
| --- | --- | --- | --- | --- | --- | --- | --- | --- | --- |
|  | Estimate | SE | t | P |  | Estimate | SE | z | P |
| Treatment (Low food vs. High food) | −0.0001 | 0.0007 | −0.215 | 0.830 |  | 0.153 | 0.096 | 1.59 | 0.111 |
| Stimulus spider (Present vs. Absent) | −0.001 | 0.0006 | −2.40 | 0.018 |  | **−0.153** | **0.039** | **−3.86** | **< 0.001** |
| Treatment:Stimulus spider | 0.0002 | 0.0003 | 0.792 | 0.430 |  | −0.045 | 0.056 | −0.805 | 0.420 |
| Body mass | **0.004** | **0.001** | **2.57** | **0.011** |  | 0.358 | 0.244 | 1.46 | 0.142 |
| Body mass:Treatment | 0.0002 | 0.002 | 0.123 | 0.902 |  | – | – | – | – |
| Body mass:Stimulus spider | **0.003** | **0.001** | **2.12** | **0.036** |  | – | – | – | – |

Note: Statistically significant P values (<0.05) are shown in boldface.

**Table S2:** Determinants of aggressiveness towards a prey stimulus (N = 864 tests on 36 focal spiders) and the web structure (N = 108 webs from 36 focal spiders).

|  | Aggressiveness | | | |  | Web structure (proportion of structural threads) | | | |
| --- | --- | --- | --- | --- | --- | --- | --- | --- | --- |
|  | Estimate | SE | z | P |  | Estimate | SE | z | P |
| Treatment (Low food vs. High food) | 1.06 | 0.913 | 1.16 | 0.613 |  | −0.352 | 0.295 | −1.19 | 0.232 |
| Stimulus spider (Present vs. Absent) | 0.375 | 0.505 | 0.743 | 0.457 |  | **0.658** | **0.240** | **2.65** | **0.007** |
| Treatment:Stimulus spider | **−1.55** | **0.705** | **−2.20** | **0.027** |  | 0.185 | 0.354 | 0.524 | 0.599 |
| Trial | **−0.102** | **0.021** | **−4.66** | **< 0.001** |  | – | – | – | – |
| Location (Between vs. Far) | **1.24** | **0.356** | **3.49** | **< 0.001** |  | ­– | – | – | – |
| Location (Near vs. Far) | **0.849** | **0.356** | **2.38** | **0.017** |  | – | – | – | – |

Note: Statistically significant P values (<0.05) are shown in boldface.

**References**

Bakdash, J.Z. and Marusich, L.R., 2017. Repeated measures correlation. Frontiers in psychology, 8, p.252904.

Schloerke, B., J. Crowley, D. Cook, F. Briatte, M. Marbach, E. Thoen, A. Elberg, and J. Larmarange. 2018. Ggally: Extension to ggplot2. R package version 1(0).
